# Increased gravitational force reveals the mechanical, resonant nature of physiological tremor

**DOI:** 10.1113/JP270464

**Published:** 2015-08-11

**Authors:** M. Lakie, C. A. Vernooij, C. J. Osler, A. T. Stevenson, J. P. R. Scott, R. F. Reynolds

**Affiliations:** ^1^ School of Sport, Exercise and Rehabilitation SciencesUniversity of BirminghamUK; ^2^ Institute des Sciences du Mouvement E.J. MareyAix‐Marseille UniversitéMarseilleFrance; ^3^ Sport, Outdoor and Exercise Science, Department of Life SciencesUniversity of DerbyUK; ^4^ QinetiQ Aircrew Systems, Air DivisionQinetiQFarnboroughUK; ^5^ Centre of Human and Aerospace Physiological SciencesKing's College LondonUK; ^6^ Wyle GmbHKölnGermany

## Abstract

**Key points:**

- Physiological hand tremor has a clear peak between 6 and 12 Hz, which has been attributed to both neural and resonant causes.
- A reduction in tremor frequency produced by adding an inertial mass to the limb has usually been taken as a method to identify the resonant component.
- However, adding mass to a limb also inevitably increases the muscular force required to maintain the limb's position against gravity, so ambiguous results have been reported.
- Here we measure hand tremor at different levels of gravitational field strength using a human centrifuge, thereby increasing the required muscular force to preserve limb position without changing the limb's inertia.
- By comparing the effect of added mass (inertia + force) *versus* solely added force upon hand acceleration, we conclude that tremor frequency can be almost completely explained by a resonant mechanical system.

**Abstract:**

Human physiological hand tremor has a resonant component. Proof of this is that its frequency can be modified by adding mass. However, adding mass also increases the load which must be supported. The necessary force requires muscular contraction which will change motor output and is likely to increase limb stiffness. The increased stiffness will partly offset the effect of the increased mass and this can lead to the erroneous conclusion that factors other than resonance are involved in determining tremor frequency. Using a human centrifuge to increase head‐to‐foot gravitational field strength, we were able to control for the increased effort by increasing force without changing mass. This revealed that the peak frequency of human hand tremor is 99% predictable on the basis of a resonant mechanism. We ask what, if anything, the peak frequency of physiological tremor can reveal about the operation of the nervous system.

**Abbreviations**
EMG: electromyography
*g*: gravitational field strength
RoG: radius of gyration
RF: resonant frequency

## Introduction

Physiological hand tremor has a very distinct peak in its acceleration spectrum. For 237 subjects, aged from 9 to 91 years, 90% had a peak between 7 and 11 Hz (Lakie, 1994). At least part of the explanation for this is mechanical (Stiles & Randall, 1967; Raethjen *et al*. 2000). The mass of the limb interacts with the elasticity of the muscles and tendons. The joint is less than critically damped so it has a resonant frequency (Lakie *et al*. 1984, 2012; Reynolds & Lakie, 2010; Vernooij *et al*. 2014). Driving input to the resonant system comes from mechanical perturbation from active postural muscles which do not produce particularly smooth output because they fire at frequencies too low for complete tetanic fusion. However, this need not be the only explanation for the tremor peak. There have been many suggestions that tremor is at least partly produced by a central or spinal neural oscillator firing at the tremor frequency (McAuley & Marsden, 2000). In this experiment we seek to show that it is not necessary to invoke such oscillators. Physiological hand tremor frequency can be adequately explained on an entirely mechanical basis. This is important because there is a widespread (and in our view, poorly substantiated) belief that physiological tremor peak frequency provides insights into the nervous system, for example ‘Tremor […] could constitute a new investigative tool providing a non‐invasive “window” into the rhythmic nature of human motor control’ (McAuley & Marsden, 2000).

**Figure 1:**
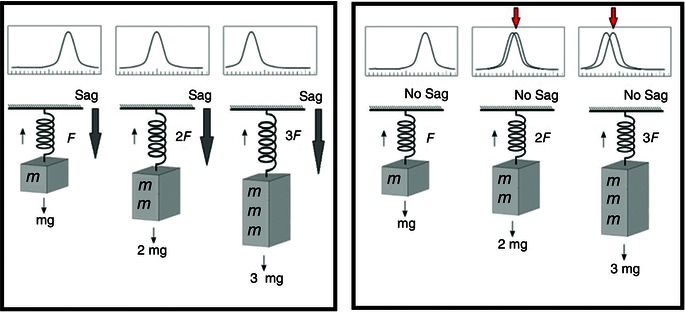
**The dual effect of added mass** A mass is supported by a spring. Resonant frequency (RF, in Hz) is dictated by spring stiffness (*k*) and by mass (*m*). The equation is RF = 1/(2π)√(*k*/*m*). Left panel: increasing mass produces increased force (*F*) which causes extension (sag) in the supporting spring if *k* does not change. The decrease in RF with added mass is indicated in the amplitude frequency spectra (top row). The reduction in resonant frequency reflects only the added mass. Right panel: this symbolises the human postural system when mass is added to a limb. In order to support the load without sag (to maintain posture), increased muscular effort is required to generate increased upward force. This muscular activity increases stiffness, and the anticipated reduction in resonant frequency is partly offset. The spectra in the top row show the spectra with sag (copied from the left panel) with the spectra with no sag superimposed (spectra are displaced to the right, indicated by arrows).

The mechanical component of tremor has generally been investigated by attaching masses (artificial inertial loads) to the limb (e.g. Marshall & Walsh, 1956; Stiles & Randall, 1967; Raethjen *et al*. 2000). The idea is that increasing the inertia will reduce the resonant frequency of the tremor in a simple and predictable way. If the tremor frequency fails to change as expected, the conclusion is drawn that other (neural) factors are (partly) responsible for the tremor. An unrecognised serious problem with this approach is that simply adding masses to the limb not only increases its inertia but also its weight. Consequently, an increase in active force, and thus electromyography (EMG), is necessary to maintain the loaded limb's position in the gravitational field. The increased muscular activity is likely to give rise to an increase in muscle stiffness, which by itself will raise the resonant frequency. As a result, the decrease in tremor frequency that occurs due to increased inertia may be (partly) counteracted by the increased force. The problem is outlined in Fig. 1 One way to overcome this problem would be to use a balanced mass in the manner of a flywheel. Balanced masses have been successfully used with a passive limb, but attaching them to a posturally active limb without compromising its movement is problematic (Lakie *et al*. 1986).

In the present experiments, which were designed to demonstrate conclusively the mechanical basis of physiological tremor, we used a large horizontal human centrifuge. By spinning subjects in the centrifuge, we were able to record naturally occurring postural hand tremor at a range of levels of gravitational field strength (*g*). While being centrifuged, the subjects had to contend with an increase in force alone. In a complementary part of the experiment, when the centrifuge was stationary, mass was added to the hand to generate inertial force loadings comparable with those produced by increased *g*. In this latter case, the subject had to contend with both increased force and the increased mass. It was thus possible to compare directly the effect of force loading and inertial loading of the limb. Subsequently, the effect produced by the increased force could be subtracted from that caused by increased mass so that the result of purely inertial loading was revealed.

## Methods

### Ethical approval

The experiments were approved by the QinetiQ Research Ethics Committee (QREC) and were conducted in accordance with standard human centrifuge operating procedures and the *Declaration of Helsinki*. Informed written consent was taken from all volunteers.

### Subjects

The experiments were performed on seven male subjects (mean age 35 years, SD 5 years) using the Farnborough (UK) human‐rated centrifuge. We intentionally only used subjects who had considerable experience in the Farnborough centrifuge in order to obviate anxiety or arousal which might be expected to increase tremor size. All subjects had previously experienced up to 9 *g* on the Farnborough machine and regarded the maximum *g* level that was used (2.5 *g*) as trivial.

### Apparatus and procedure

**Figure 2:**
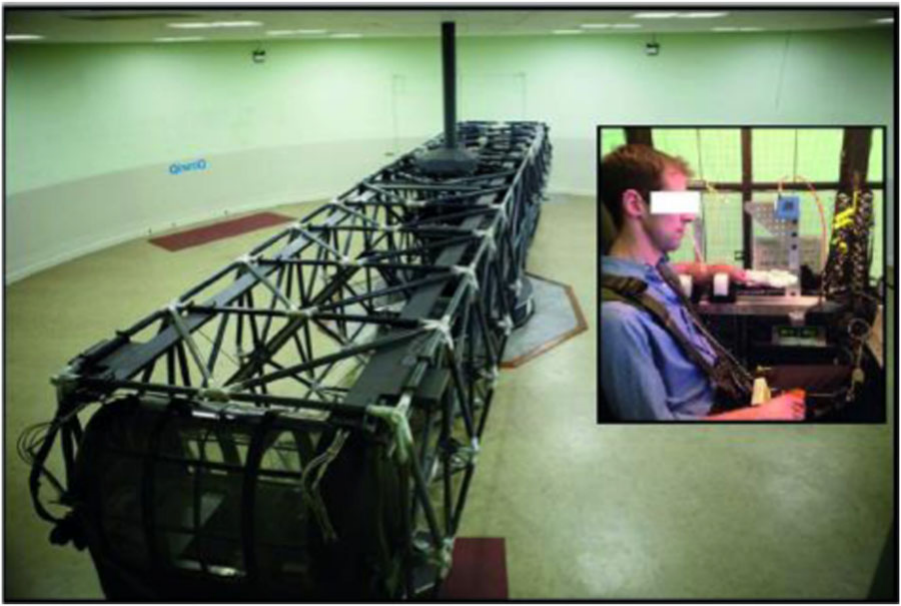
**Experimental setup** To the left of the picture is the centrifuge with one of its two pivoted gondolas at the end of its ∼10 m radius arm. The inset shows a subject strapped into the seat. A thermoplastic splint ensured the hand and fingers moved as one. A laser rangefinder situated above the hand was used to monitor its vertical position, which was displayed to the subject on a screen just out of shot on the right of the picture. The accelerometer is located on top of the hand splint but is too small to be seen distinctly. The EMG electrodes lie underneath the protective bandaging on the forearm.

The SI abbreviation for local gravitational field strength is *g* and for mass is kg, which prevents unintentional confusion. Figure 2 depicts an instrumented subject seated in the centrifuge. A lightweight moulded splint (thermoplastic, 0.1 kg), which held the digits in a slightly adducted and fully extended posture, was attached to the subject's hand. A 3‐axis accelerometer (ADXL335; Analog Devices, Norwood, MA, USA) was firmly attached to the dorsum of the splint to record hand tremor. Throughout the experiment, subjects sat in the gondola at the end of the centrifuge arm (approximately 10 m from the axis of rotation, and were harnessed into an aircraft pilot's seat (Mk 16; Martin Baker Aircraft Company Ltd, Higher Denham, Middlesex, UK) with the pronated left forearm supported by an armrest and foam splinting at approximately chest level. The subject was asked to hold his hand in a horizontal position. A retroreflective laser rangefinder (Model YP11MGV80; Wenglor sensoric GmbH, Tettnang, Germany) was focused on the dorsum of the hand in order to monitor hand position and this signal was recorded and displayed to the subject on a monitor screen at eye level at ∼1 m distance. The gondola was hinged so that the effective acceleration vector always passed precisely in the head‐to‐foot (typically referred to as +Gz) direction (Latham, 1955). In the first part of the experiment, the centrifuge was rotated at angular velocities from zero to a maximum of approximately 15 r.p.m. to generate forces of 1, 1.5, 2 and 2.5 *g*. The onset and offset rate of change of acceleration was 1 *g* s^−1^ and the subjects were spun at the designated velocity for 45 s; the first and the last 1 s of the steady state record were discarded from analysis. Each subject performed three repeats of each designated velocity. To establish a ‘noise floor’ and control for minor accelerations produced by small changes in angular velocity of the centrifuge or vibration, acceleration was recorded while the accelerometer was attached to an inanimate object in the capsule. This broadband noise was two orders of magnitude less than the tremor signal at the highest (noisiest) rotation velocity. In addition to hand tremor and position we recorded surface EMG from the extensor digitorum communis and flexor digitorum superficialis muscles using a two channel Delsys Bagnoli system (Delsys Inc., Boston, MA, USA). As part of routine centrifuge subject monitoring, limb lead ECG and blood pressure (Portapres, Finapres Medical Systems, Amsterdam, The Netherlands) were also recorded.

In the second part of the experiment, tremor was recorded with masses attached to the hand while the centrifuge remained stationary. These were made from lead sheet and had masses of 0.250 kg, 0.500 kg and 0.750 kg. Each subject performed three repeats with each mass. Because the average mass of the instrumented hand is approximately 0.500 kg and the weights were positioned as closely as possible above the centre of mass of the hand, the subject had to exert an upward force approximately equivalent to that experienced at 1.5 *g*, 2 *g* and 2.5 *g*. The hand mass for each subject was calculated using a simple anthropometric model and a scanned and dimensioned image of the hand (for details see Appendix). To enable direct comparison between the two conditions (increased gravity and increased mass), we present data recorded at four equivalent levels, called simply ‘load’ (Table 1). All data were sampled at 1 kHz. The hand would more correctly be considered as a torsional oscillator with the wrist having an angular stiffness and the hand having a moment of inertia (Lakie *et al*. 1986). For simplicity, and following common practice, we used a linear approximation where the mass of the hand is suspended by muscles and tendons with an elastic stiffness. This approximation does not introduce much inaccuracy and we explain our reasoning in the Appendix.

**Table 1:** **Loads applied in two conditions (altered gravitational field (*g*) and added mass (kg))**

Load	Gravitational field(*g*)	Added mass(kg)
1	1	0
2	1.5	0.250
3	2	0.500
4	2.5	0.750

### Analysis

Analysis was performed by custom‐written programs in MATLAB software (Matlab 2011*a*; MathWorks, USA). EMG was band‐pass filtered between 30 and 200 Hz and rectified. For each trial, the amplitude spectra of the hand acceleration and EMG were obtained by NeuroSpec software (version 2.0, 2008, http://www.neurospec.org/) and the maximum amplitude and associated frequency were detected. For all subjects, the resulting data (maximum amplitude and associated frequency) were then averaged, including the three trials for each condition. Repeated‐measures ANOVA was used to test for significant effects of load. *P* < 0.05 was considered significant.

## Results

### Tremor acceleration and frequency

**Figure 3:**
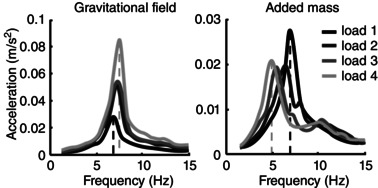
**Representative subject data** Tremor acceleration spectra when load is increased in the centrifuge (left panel) or by adding mass (right panel). With added gravitational field strength the tremor size increases and the frequency rises somewhat. With added mass, the tremor size decreases slightly and the frequency falls.

The results from a typical subject are shown in Fig. 3 With increasing *g* (left panel) tremor acceleration increased progressively from its baseline level until it became approximately four times bigger. The peak frequency also increased progressively, in this case from ∼6.5 Hz to ∼7.5 Hz. The effects of increasing mass (right panel) were opposite: tremor size decreased and the frequency fell, in this case from ∼6.5 Hz to ∼5 Hz. These results were typical of all subjects and Fig. 3 shows the mean (±SEM) frequency for both conditions.

In this study we are mainly interested in the peak frequency of tremor. However, with added mass, the frequency spectrum in this representative subject also shows typically variable sub‐peaks at higher frequencies (10–20 Hz) which are close to those seen in the EMG (Fig. 6).

**Figure 4:**
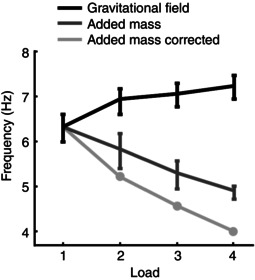
**Group mean tremor frequency** The tremor peak frequency (mean and SEM) in both conditions; increased *g* (black) and increased mass (dark grey) for each load. The corrected values for each load (decrease caused by mass minus increase caused by *g*) are also plotted (light grey).

Figure 4 shows the progressive decrease in tremor frequency as inertia is increased and the progressive increase in tremor frequency as *g* is increased (*F*_3,18_ ≥ 15.9; *P* < 0.001). The decrease in frequency with added mass is the expected effect of adding mass to a spring mass system. The rise in frequency with added *g* is simply explained as the result of increased limb stiffness as a result of increased muscle activity required to support the hand. Increasing *g* has no direct effect on the resonance of the spring and mass. Accordingly, it now becomes possible to correct for the inevitable increased stiffness in the mass loading condition. In Fig. 4 this has been done by piecewise correction at each load condition. For example, the effect of mass at load 4 has been to lower the resonant frequency by approximately 1.4 Hz. However, this mass loading will have also produced an increase in resonant frequency of approximately 0.9 Hz because of necessary muscular stiffening and this will have partly offset the reduction caused by the added mass. Correction reveals the true reduction (approximately 2.3 Hz) that would be produced by solely the mass. We can use piecewise correction for each condition, since the added masses were chosen to match each *g* level.

**Figure 5:**
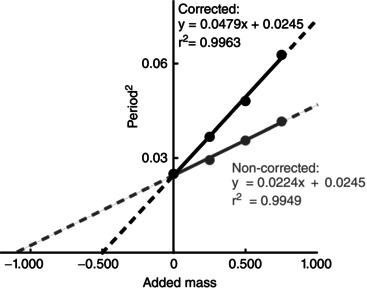
**Estimated hand mass** The corrected values and non‐corrected values of the added mass condition from Fig. 4 are plotted as oscillation period (Period) squared *vs*. added mass. Both lines provide a very good fit to the data points. Extrapolation to the point where the regression line cuts the *x*‐axis yields the amount of mass that would have to be removed to reduce the Period to zero – that is, the mass of the hand and splint. The uncorrected values predict a mass of ∼1.1 kg, whereas the corrected values predict a mass of ∼0.5 kg.

There are two lines of evidence that suggest that we can directly compare the two conditions. First, we can use the data from Fig. 4 to calculate the mass of the hand and compare this to our estimation of hand mass based on hand size (see Appendix). Addition of mass to a resonant system will produce a linear increase in the period of oscillation (‘Period’) squared (Lakie *et al*. 1986). Backwards extrapolation of this relationship will reveal the notional mass that has to be removed to make the period zero – that is, the original mass of the limb (plus splint). This relationship is shown in Fig. 5 and further described in the Appendix.

**Figure 6:**
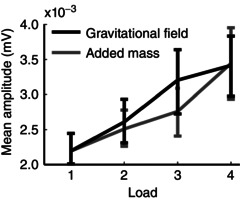
**Mean filtered rectified extensor EMG at each load (±SEM)**

In Fig. 5 the frequency values obtained from the condition where mass is added have been replotted in terms of Period^2^. Using uncorrected frequency values from Fig. 4 (dark grey traces) implies a mass of 1.09 kg for the hand plus splint, whereas the use of corrected values (light grey traces) implies a mass of 0.493 kg. These values may be compared with the retrospectively estimated mass of our subjects’ hands (see Appendix) which was 0.460 kg. The agreement when the corrected values are used is close. The second line of evidence comes from the EMG, because we can compare whether the amount of EMG activity required to support different loads was similar in the two conditions. We used a standard added mass which assumed that the mass of the hand and splint was ∼0.500 kg. Therefore the effect of adding a mass of 0.500 kg should double the effort, and thus EMG, required to support the hand. Increasing the gravitational field strength to 2 *g* should have the same effect. The close correspondence between the two conditions is shown in Fig. 6 Addition of mass or *g* both similarly increased EMG (*F*_3,18_  ≥ 11.3; *P* < 0.001).

Figure 6 shows that there is a very good agreement between the sizes of EMG at each load condition. This figure therefore also strongly supports the assumption that the mass of the hand plus splint is close to 0.500 kg.

**Figure 7:**
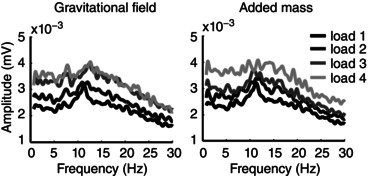
**Mean extensor EMG spectra** Left panel shows the effect of increasing *g*. Right panel shows the effect of increasing mass. At each load the overall level of EMG is similar and the spectra all have a similar shape. It is clear that in the higher *g* conditions, but not in any of the other conditions, there is an emergent small peak close to the frequency of the tremor.

The increase in tremor frequency with increased *g* was shown in Fig. 4 and we have attributed it to increased muscular stiffness contingent on the effort. Are there other possible reasons why tremor frequency might increase under conditions of increased gravitational field strength? A possible candidate would be that it is due to a progressive, gravity dependent change in EMG frequency. This is examined in Fig. 7 which shows the mean rectified extensor EMG spectra in each condition.

Three features are clear in Fig. 7 First, the general shapes of the spectra are always similar, with a broad peak between 8 and 14 Hz in each load condition which does not change systematically with load. Second, the sizes of the spectra are well matched between added *g* and added mass for each load condition, further supporting the statement that gravitational and mass loads are equivalent. Third, there is a small sub‐peak in EMG activity at the approximate frequency of the tremor in the increased *g* conditions. It is noteworthy that these are the conditions in which the tremor acceleration is particularly large.

**Figure 8:**
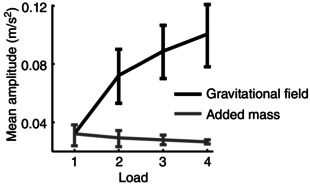
**Mean amplitude of peak tremor acceleration at each load (±SEM)**

In addition to the effect on frequency, increasing the load on the hand also affected the size of tremor. The tremor acceleration (size) was shown for an individual subject in Fig. 3 and is shown for all subjects in Fig. 8.

Figure 8 shows that increasing gravitational field produces a large increase in tremor acceleration (*F*_3,18_ = 11.7; *P* < 0.001). Acceleration is related to force, which rises progressively as *g* increases. The effect is progressive but not quite linear. Conversely, increasing the mass produced no significant change (*F*_3,18_ = 0.53; *P* = 0.67).

## Discussion

We discuss our results under five headings.

### (1) The effect of increased mass

In our experiments, the addition of mass systematically decreased the frequency of tremor (Fig. 4). There is some disagreement in the literature about the effect of added mass on tremor frequency. Marshall & Walsh (1956) found that frequency did not change. Hamoen (1962) was the first to show that human tremor frequency was decreased by added mass. This was subsequently confirmed by Stiles & Randall (1967), and confirmed in a reduced animal model (Rietz & Stiles, 1974). However, in a subsequent study (Stiles, 1980) the author found that the effect of added mass was variable and depended on the posture of the hand.

When a decrease in tremor frequency occurred, it has always been attributed to a reduction in frequency of a mechanically resonant limb. It is difficult to think of any alternative explanation. A load sensitive neural oscillator is one possibility, but it would have to possess the feature that its frequency of operation decreases as limb mass increases. *Prima facie* this seems unlikely. There is no peak in the EMG at the tremor frequency (Fig. 7 right panel). Additionally, this figure shows that, if anything, as mass is added, there is a slight increase in the frequency where the rectified EMG is largest. Neural drive that shifts to a lower frequency with added mass thus seems highly unlikely. Furthermore, we show in these experiments that increasing the load, and thus EMG, by increasing *g* is associated with a clear *rise* in tremor frequency. Therefore, the most likely cause of the reduction in tremor frequency with added mass is a reduction in the resonant frequency of the limb. Furthermore, our experiments provide a natural explanation for the apparently capricious response to increased mass described by other authors, above. As mass produces an inevitable increase in stiffness its effect will depend on whether the increased inertia (lowering frequency), or the increased stiffness (raising frequency), prevails. In turn, this will depend on the precise detail of how the procedure is performed. Because load is a linear function of the moment arm but inertia depends on the square of the radius of gyration, much will depend on where the additional mass is positioned (see Appendix).

### (2) The effect of increased gravitational field strength

Increasing *g* increased the frequency of tremor (Fig. 4). This change has not been previously described and seems to have only two possible explanations. It is conceivable that there is an increase in the frequency of a putative tremor oscillator as gravitational load increases. Note that it has to be gravitational load because increasing load by the addition of mass is associated with a reduction of tremor frequency as described above. Such a specifically gravity sensitive oscillator seems highly unlikely. It is undeniable that in Fig. 7 there is some prominent EMG activity close to the tremor frequency as *g* is increased. While this might possibly support the notion of a tremor oscillator which is uniquely sensitive to gravitational load it seems much more likely that it actually reflects some reflexive modulation of EMG activity by limb motion under conditions where tremor is particularly large. From Fig. 8 it can be seen that tremor is approximately four times larger at 2.5 *g* compared to 1 *g*. Modulation of EMG in enhanced tremor has been previously described (Hagbarth & Young, 1979) and is probably an inevitable consequence of reflex modulation of motor output driven by muscle spindles or other afferents. There is no reason to suppose that increased depth of modulation as tremor size increases will automatically produce an associated increase in frequency. However, a natural explanation for the elevated tremor frequency in increased *g* is that increased muscular effort leads to inevitable stiffening of the muscles. Many studies have shown an activity dependent increase in skeletal muscle stiffness (Joyce & Rack, 1969; Cannon & Zahalak, 1982; Kearney & Hunter, 1990). Although this stiffening will be mitigated to some degree by the compliance of the series tendons, it will be sufficient to increase the resonant frequency and tremor frequency will rise. Resonant frequency squared is proportional to stiffness. Figure 4 suggests that in going from 1 *g* to 2.5 *g* the stiffness increases by approximately 25%. As we show below, increased wrist stiffness provides an almost perfect explanation for our results. We studied the effect of increased *g*. However, our results can also be used to predict the effect of reduced *g* and we describe this relationship in the Appendix.

### (3) The effect of the combination of increased mass and increased stiffness

As described in section (1) the addition of mass decreases resonant frequency of a limb. However, as described in section (2) the increased muscular effort as mass is added will cause a degree of increased limb stiffening and therefore an increase in resonant frequency. Because these two changes will occur simultaneously, the increased stiffness will partly offset the reduction in frequency caused by the mass. The extent of this offset is revealed in Figs 4 and 5. In these figures we have plotted the reduction in resonant frequency caused by added mass when the increased stiffness is both ignored and included. In Fig. 5 we have plotted this in terms of Period^2^ plotted against added mass. By doing this, it is possible to deduce the mass of the hand and the stiffness of the wrist (see Appendix). The use of uncorrected values leads to an estimate of > 1.0 kg for hand mass and > 1700 N m^−1^ for wrist stiffness. When the values are corrected by piecewise correction of the increased frequency produced by muscular stiffening, these estimates have much more realistic values (0.493 kg and 793 N m^−1^). The estimated mass includes 0.1 kg for the mass of the hand splint so our predicted hand mass is 0.393 kg. This value is very close to the retrospectively measured mean hand mass for our subjects: (0.360 kg) (Appendix).

There are several published estimates for the mass of the human hand. The classic cadaveric study by Clauser *et al*. (1969) gives values of 0.380 kg, 0.446 kg and 0.548 kg for small, medium and large male subjects. Drillis *et al*. (1964) tabulate hand mass measurements as a percentage of body mass found by other investigators. From their Table 6, estimated hand mass for a 70 kg man would be 0.420–0.588 kg. Our subjects may have been slightly smaller than average, but unfortunately we do not have data for their height or weight. However, the main point is clear. Hand mass, even for the largest subjects, will not exceed 600 g. Only corrected values predict a hand mass of anything close to a realistic estimate. It is absolutely necessary to include the increased stiffening.

Furthermore, the gradients of the regression lines in Fig. 5 reflect stiffness (details in Appendix). The use of uncorrected values in our experiment predicts a stiffness of 1761 N m^−1^ whereas corrected values predict a stiffness of 793 N m^−1^. There are several published estimates of human wrist stiffness for flexion/extension. A complication is that these values are often expressed in angular units, that is, as N m rad^−1^. In order to convert to linear units as used in our study it is necessary to know the moment arm of the wrist for flexion/extension. A sensible estimate for this is 1.3 cm (see Figs 3 and 4 in Gonzalez *et al*. 1997). When published values are adjusted in this way a definitive study on 10 subjects by Halaki *et al*. (2006) gives a mean value for wrist stiffness of 534.8 N m^−1^ (range 320.7–1015.4 N m^−1^; their Table 3). Clearly, our uncorrected stiffness values are impossibly high whereas the corrected values are in the appropriate range. There is also the fact that had we used a possibly more appropriate torsional model of the hand the equivalent masses would have been applied at the radius of gyration rather than the centre of mass. Had we done so there would have been a greater reduction in resonant frequency and our stiffness estimate would reduce further bringing them even closer to published values (details in Appendix).

These results imply that, to generate realistic estimates of hand mass and wrist stiffness, it is essential to include the increased stiffness that is a consequence of increased load. When this is done, tremor frequency provides very robust estimates of limb mass and stiffness and this is very strong evidence that the hand tremor frequency results from resonance only. The coefficients of determination of the regression lines of Fig. 5 show that more than 99% of the change in frequency can be explained by a simple spring/mass oscillator. It is common to describe physiological tremor by the frequency of its spectral peak, for example, 8–12 Hz tremor or ∼10 Hz tremor. What is clear from our experiments is that the peak frequency of tremor says a lot about the stiffness and mass of the hand and wrist, but little about the neural input. The EMG spectrum is a clear reflection of the output of the CNS but the acceleration spectrum of tremor is mainly determined by mechanical factors. The peak frequency of the physiological tremor we recorded was greatly altered by changed loads whereas the EMG was not. Thus tremor frequency revealed much more about the load than about neural oscillators, central or spinal. From these results it is difficult to believe that the study of physiological peak tremor frequency can show anything more than the mechanical features of the musculoskeletal system. It does not provide an insight into neural rhythmicity (McAuley & Marsden, 2000). However, because tremor frequency does reflect the stiffness of the muscles it can provide a useful insight into their state (Vernooij, 2014).

### (4) Comparison of EMG levels for each load condition

Central to our approach was the belief that the mass of the instrumented hand was close to 0.500 kg. This is supported by our own and other estimates (see Appendix and section (3) above). However, this assumption also underpinned our belief in the equality of the mass and gravitational loading. The equality is confirmed by Figs 6 and 7, which show that very similar levels of EMG were associated in both conditions with the corresponding load so that, for example, the effect of the addition of 0.500 kg was the same as doubling the gravitational field strength. The shape of the EMG spectrum always had a very broad peak of activity around 8–14 Hz, which was not close to the tremor frequency. This feature of the EMG has been frequently described (Timmer *et al*. 1998; Halliday *et al*. 1999; Raethjen *et al*. 2000). EMG spectra are similar in both conditions at each load level and the only observable difference is a small peak close to the tremor frequency when the tremor is particularly large (discussed in section (2) above).

### (5) Tremor size increased considerably with increased *g* and decreased slightly with added mass

Tremor acceleration represents force fluctuation which is approximately proportional to background force (signal dependent noise; Schmidt *et al*. 1979; Slifkin & Newell, 1999; Enoka *et al*. 1999; Laidlaw *et al*. 2000). Because of Newton's second law (*A* = *F*/*M*), as force fluctuations increase, tremor acceleration becomes larger. Consequently, an increase in tremor size with *g* is anticipated. Figure 8 shows that the relationship is not quite linear. A likely explanation is that limb damping also increases with activity and this acts to attenuate the tremor as *g* is increased. With mass loading, because *A* = *F*/*M*, as mass is increased acceleration is decreased. However, this is largely offset by the associated increase in force fluctuation as load increases. It is interesting that Fig. 8 shows that tremor acceleration tends to decrease a little as mass is added although the reduction does not reach statistical significance. The implication is that the effect of added mass may outweigh the increased force fluctuations.

### Conclusion

The effects on physiological tremor of adding inertia to a limb can only be explained if the concomitant changes in stiffness are taken into consideration. Peak tremor frequency reflects the inertia and stiffness of the limb and is not the result of a central oscillator. These results are restricted to physiological tremors. In most pathological tremors the tremor rhythm is probably dominated by neural drive at a specific frequency; however, even in that situation, mechanical factors will play an inevitable role.

## Additional information

### Competing interests

The authors have no competing interests to declare.

### Author contributions

Authors are presented in alphabetical order. M.L., R.F.R., C.A.V.: conception of the experiments. M.L., C.J.O., R.F.R., A.T.S., J.P.R.S., C.A.V.: experimental design and collection and assembly of data. M.L., R.F.R., C.A.V.: analysis and interpretation of data. M.L., R.F.R., C.A.V.: drafting the article and revising it critically for important intellectual content. All authors approved the final version of the manuscript, all persons designated as authors qualify for authorship, and all those who qualify for authorship are listed. The experiments were performed at the human centrifuge facility, QinetiQ, Farnborough. UK.

### Funding

This work was funded by a BBSRC Industry Interchange Award to J.P.R.S. and R.F.R. C.J.O. was funded by BBSRC grant BB/I00579X/1. C.A.V. was funded by A*Midex (Aix‐Marseille Initiative of Excellence).

**Translational perspective**

Physiological tremor has been studied for over a century but the mechanisms remain contentious. Two explanations are generally proffered. Firstly, that oscillations within the central nervous system manifest as movement, and secondly, that tremor is simply the consequence of the mechanical properties of the limb. Here we attempt to distinguish between these explanations by changing the mechanical properties of the hand and observing the consequences upon tremor. In one case, we added mass to the hand to increase its inertia. This reduced tremor frequency, but not quite as much as predicted by a purely resonant mechanical system. However, adding mass also increased the required muscular effort to hold the hand against gravity, potentially increasing joint stiffness and thus resonant frequency. To account for this effect, we devised a situation whereby increased muscular effort was necessary, but without any change in inertia. This was achieved by using a human centrifuge to increase gravitational acceleration (*g*). We observed that, unlike added mass, higher *g* did indeed cause an *increase* in tremor frequency. By matching the *g* level to the amount of added mass in the previous condition, we were able to effectively subtract the effect of muscular effort. When we did this, the change in tremor frequency was entirely explicable on the basis of a mechanical resonant system. This suggests that physiological tremor primarily reflects the physical properties of the limb in question, rather than its neural input.

## Figures and Tables

**Figure 9:**
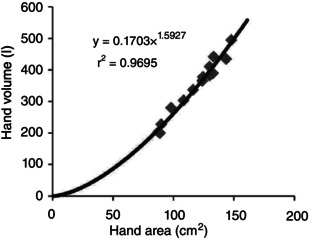
**The relationship between area of the hand and volume for 14 subjects** The equation of the regression line was used subsequently to estimate the hand volume of the 7 different subjects that we used in the centrifuge experiments.

**Figure 10:**
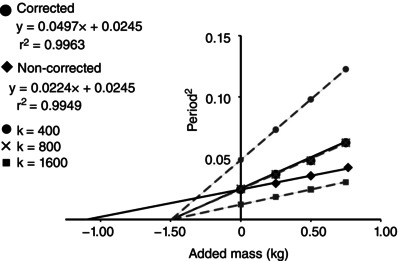
**The relationship between oscillation period squared and added mass for a resonant system**

**Figure 11:**
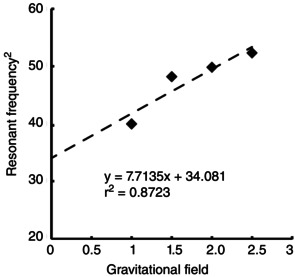
**The relationship between resonant frequency squared and *g***

## Calculation of hand mass

Ideally, we would have measured the volume of each subject's hand at the time of making the tremor measurements, but this was not possible. We were able to obtain high quality 2‐D scans of each subject's hand and the surface area of this scan was used as the input to an equation which we subsequently derived from a separate group of 14 subjects with a broad range of hand sizes. This equation defined the relationship between hand surface area and hand volume and was calculated in the following way. Subjects placed their left hand, palmar side down, on the surface of a scanner. The resultant image was processed using an edge detection algorithm (define hand edges: Adobe Photoshop; convert edges to binary *x–y* coordinates: ImageJ; Schneider *et al*. 2012) which outlined the hand. The hand surface was defined as the area bounded by this margin and the line of the radiocarpal joint. Hand volume was measured by water displacement when the hand was immersed up to the line of the radiocarpal joint. The relationship between measured volume and hand surface area was *y* = 0.1703*x* ^1.5927^ (*r* ^2^ = 0.9695; see Fig. 9). Note that this proportionality is very similar to the surface–volume relationship of a regular rectangular object; i.e. *x* ^1.5^. This equation was then used to estimate the hand volume of the subjects who participated in our study (see Table 2). These volumes were finally converted to mass using a value for density of 1.1, which reflects the preponderance of bone in the hand. This value lies approximately midway between two previously reported values for hand density. (Harless, 1860, cited in Drillis *et al*. 1964) gave a value of 1.1128 and, more recently, Clarys & Marfell‐Jones (1986) gave a value of 1.0823 (our calculation of overall hand density from their Table 3).

**Table 2:** **Subject hand parameters**

	Hand	Hand	Mass	Hand	Centre of mass
Subject	area(cm^2^)	volume(l)	(kg)	length(cm)	(cm from axis)
1	109.7	0.302	0.332	18.4	8.24
2	91.4	0.226	0.249	17.3	7.73
3	130.1	0.397	0.437	20.5	9.16
4	131.4	0.403	0.443	20.0	8.96
5	106.1	0.287	0.316	18.5	8.29
6	127.5	0.384	0.422	20.7	9.25
7	107.6	0.293	0.322	17.7	7.91
Mean	114.8	0.327	0.360	18.7	8.64

**Table 3:** **Prediction of human hand tremor frequency in non‐terrestrial gravitational environments**

Environment	*g*	RF
Space station	∼0	5.830952
Moon	0.16	5.93579
Mars	0.38	6.076989
Earth	1	6.458328
Jupiter	2.5	7.298973

*g*, gravitational field strength; RF, resonant frequency.

There are, unsurprisingly, few relevant studies directly measuring hand mass. Clauser *et al*. (1969) give values of 0.380 kg, 0.446 kg and 0.548 kg for the cadaveric hands of small, medium and large male subjects. Clarys & Marfell‐Jones (1986) found a mean hand mass of 0.345 kg for three male and three female subjects. The estimated measured volumes of our 14 subjects (Fig. 9) ranged from approximately 0.200–0.500 l implying a mass of 0.220–0.550 kg, which is similar to the above values. The mass of the splint plus accelerometer (0.100 kg) must be added to the calculated hand masses of the subjects who took part in the experiment.

The weights that we used were designed so that subjects when loaded would have to exert an upwards force nominally equivalent to that generated when they were centrifuged at 1.5, 2 and 2.5 *g*. These weights had to be prepared in advance of the experiment. We used an estimated hand mass for our subjects of 0.400 kg. The estimate was appropriate; the subsequently calculated mean hand mass of the subjects that we used in the centrifuge was 0.360 kg (Table 2). For both conditions, i.e. force and inertia, the mass of the splint plus accelerometer (0.100 kg) must be added to the calculated hand masses. This meant that the total mass became 0.460 kg, which was very close to the value which we had assumed would produce equal mass and gravitational loading (0.500 kg).

The relationship between the hand mass and the added moment of inertia we wanted to apply with the weights is quite complex because it depends on the shape as well as the size of the hand. Using data from de Leva (1996) the centre of mass was individually located at 44.8% of the hand length, defined as the distance between the radiocarpal joint and dactylion (see Table 2). When mass was added to the hand it was applied as closely as possible above this point. In doing this we reproduced what is usually done in ‘added inertia’ tremor experiments because we used a linear approximation of a torsional system. Technically, we should have applied loads which were multiples of hand mass at the radius of gyration (RoG). We did not do this for four reasons. (a) There are very few estimates of moment of inertia of the hand and this is required to calculate the RoG (RoG^2^ = Moment of Inertia/Mass). (b) We followed common practice in ‘added load’ tremor experiments. (c) The difference is probably quite small. Winter (2009) gives 51% of hand length for the centre of mass (CoM) and 59% for RoG. (d) The splint that we used also adds some inertia. We mention the possible size of the resultant error below.

## The relationship between added inertia and resonant frequency

The relationship (when frequency is measured in Hz) between resonant frequency (RF), mass (*m*) and stiffness (*k*) is

$$\mathrm{RF}=\;\frac{1}{2\pi }\;\sqrt{\;\frac{k}{m}}$$

When mass is added to a resonant system its resonant frequency is decreased. The relationship was described by Lakie *et al*. (1986). The relationship between added mass and oscillation period (Period) squared is linear, and the *x*‐intercept indicates the notional mass that must be removed to reduce the mass to zero: that is, the original mass of the system. When resonant frequency is plotted in terms of Period^2^ against added mass the result is a straight line with the equation:

$$y=\;\frac{{\left(2\pi \right)}^{2}}{k}x+\frac{{\left(2\pi \right)}^{2}}{k}m$$

for *y* = 0

$$0=\;\frac{{\left(2\pi \right)}^{2}}{k}x+\;\frac{{\left(2\pi \right)}^{2}}{k}m$$

Consequently, when *y* = 0, *m* = −*x*. Stiffness (*k*) is the reciprocal of ‘gradient’/(2π)^2^. This relationship is explored in Fig. 10.

In Fig. 10, as described above, the *x*‐intercept represents the original mass and the gradient reflects the stiffness of the system. The experimental results are fitted by continuous regression lines. For corrected results (black circles) this line (*r* ^2^ = 0.996) indicates an original mass of 0.493 kg and a stiffness of 793 N m^−1^. To indicate the sensitivity of the system to stiffness, the figure also shows modelled results for the mass of 0.493 kg stabilised by three different values of stiffness (squares, 1600 N m^−1^; crosses, 800 N m^−1^; and circles, 400 N m^−1^, all grey and fitted by dashed regression lines). Clearly, the stiffness that satisfies our corrected experimental results cannot be very far away from 800 N m^−1^. When the uncorrected results are plotted (black diamonds) they are also well fitted by a linear regression line (*r* ^2^ = 0.995), but this line predicts an original mass of 1.09 kg and a stiffness of 1760.9 N m^−1^, both unfeasibly large values.

If the hand is considered as a torsional oscillator, to double the moment of inertia a mass equivalent to the hand should have been placed at the RoG. Because we placed the equivalent mass at the CoM, which is more proximal (45% rather than 59% of hand length) (Winter, 2009), we have possibly added less inertia than we claim and not produced as big a reduction in frequency as we should have. With a greater reduction in frequency there would have been a bigger increase in Period^2^ in Fig. 10. Correcting for this difference gives a ∼28% lower value of stiffness which would move our value to 571 N m^−1^, even closer to those calculated by Halaki *et al*. (2006). However, this is partly offset by the additional moment of inertia added by our splint. With the uncertainties about the precise location of RoG, load positioning, splint inertia and the conversion factor used to convert angular into linear wrist stiffness we do not wish to overstate this claim, but it is much more likely that we have overestimated, rather than underestimated, stiffness.

## The effect of reduced *g*

The relationship between gravitational field strength and tremor frequency is restated in Fig. 11. Because, as explained, the effect of increased gravity is to alter stiffness there is a linear relationship between *g* and tremor frequency squared.

This suggests the potentially testable hypothesis that human hand tremor frequency will be different in non‐terrestrial gravitational environments. The equation is

$$\mathrm{RF}=\;\sqrt{7.7g+34}$$

where *g* is gravitational field strength relative to Earth and resonant frequency (RF) is in Hz.

This predicts the values in Table 3.
